# Recovering high-quality bacterial genomes from cross-contaminated cultures: a case study of marine *Vibrio campbellii*

**DOI:** 10.1186/s12864-024-10062-2

**Published:** 2024-02-06

**Authors:** Neža Orel, Eduard Fadeev, Gerhard J. Herndl, Valentina Turk, Tinkara Tinta

**Affiliations:** 1 Marine Biology Station Piran, National Institute of Biology, Piran, Slovenia; 2 Department of Functional and Evolutionary Ecology, Bio-Oceanography and Marine Biology Unit, University of Vienna, Vienna, Austria; 3 NIOZ, Department of Marine Microbiology and Biogeochemistry, Royal Netherlands Institute for Sea Research, Den Burg, The Netherlands

**Keywords:** Whole-genome assembly, Non-axenic culture, *Vibrio campbellii*, Plasmid, Marine bacteria

## Abstract

**Background:**

Environmental monitoring of bacterial pathogens is critical for disease control in coastal marine ecosystems to maintain animal welfare and ecosystem function and to prevent significant economic losses. This requires accurate taxonomic identification of environmental bacterial pathogens, which often cannot be achieved by commonly used genetic markers (e.g., 16S rRNA gene), and an understanding of their pathogenic potential based on the information encoded in their genomes. The decreasing costs of whole genome sequencing (WGS), combined with newly developed bioinformatics tools, now make it possible to unravel the full potential of environmental pathogens, beyond traditional microbiological approaches. However, obtaining a high-quality bacterial genome, requires initial cultivation in an axenic culture, which is a bottleneck in environmental microbiology due to cross-contamination in the laboratory or isolation of non-axenic strains.

**Results:**

We applied WGS to determine the pathogenic potential of two *Vibrio* isolates from coastal seawater. During the analysis, we identified cross-contamination of one of the isolates and decided to use this dataset to evaluate the possibility of bioinformatic contaminant removal and recovery of bacterial genomes from a contaminated culture. Despite the contamination, using an appropriate bioinformatics workflow, we were able to obtain high quality and highly identical genomes (Average Nucleotide Identity value 99.98%) of one of the *Vibrio* isolates from both the axenic and the contaminated culture. Using the assembled genome, we were able to determine that this isolate belongs to a sub-lineage of *Vibrio campbellii* associated with several diseases in marine organisms. We also found that the genome of the isolate contains a novel *Vibrio* plasmid associated with bacterial defense mechanisms and horizontal gene transfer, which may offer a competitive advantage to this putative pathogen.

**Conclusions:**

Our study shows that, using state-of-the-art bioinformatics tools and a sufficient sequencing effort, it is possible to obtain high quality genomes of the bacteria of interest and perform in-depth genomic analyses even in the case of a contaminated culture. With the new isolate and its complete genome, we are providing new insights into the genomic characteristics and functional potential of this sub-lineage of *V. campbellii*. The approach described here also highlights the possibility of recovering complete bacterial genomes in the case of non-axenic cultures or obligatory co-cultures.

**Supplementary Information:**

The online version contains supplementary material available at 10.1186/s12864-024-10062-2.

## Background

Coastal ecosystems are subject to various natural perturbations (e.g., variations of physical, chemical and biological conditions) and increasing anthropogenic pressures (e.g., overpopulation of coastal areas, mariculture, agriculture, maritime traffic). This creates conditions in which allochthonous human pathogens, e.g., introduced via wastewater, ballast water or coastal runoff, and indigenous marine animal pathogens are likely to thrive [1]. As coastal waters are used for recreation and food production, the occurrence of pathogens can have a direct high economic and social impact [2]. Fast and accurate surveillance of potential pathogens is therefore crucial to predict the risk of disease outbreaks and to understand disease-promoting environmental conditions.

Advanced molecular approaches and next-generation sequencing (NGS) led to the widespread use of culture-independent monitoring methods, such as high throughput sequencing of marker genes (i.e., amplicon sequencing) [3]. However, in the case of many bacterial pathogens, these approaches are not sufficient for their accurate identification. The decreasing costs of whole genome sequencing (WGS) and the development of new bioinformatics tools for genomic analyses provide new opportunities not only to accurately detect pathogens, but also to gain valuable insights into their functional potential [4–6]. Whole genome analyses were successfully applied in epidemiological studies, revealing sources, means of transmissions, and outbreak dynamics of non-marine bacterial pathogens [7, 8]. Detecting pathogens at different spatial-temporal scales in different ecosystems and analyzing their functional potential using their complete genomes can provide answers to important ecological questions, such as adaptation to different ecological niches, pathogen-host interactions and dispersion of functional genes between different strains [9, 10].

The long-established approach of obtaining a pure (axenic) culture of the strain of interest, followed by DNA extraction and high-throughput sequencing, is still probably the best way to obtain a high-quality bacterial genome [11]. However, obtaining an axenic bacterial culture from environmental samples is often challenging since contamination can occur during any of these steps, even when strict microbiological standards and aseptic techniques are applied [12, 13]. Therefore, non-axenic cultures represent a practical challenge to obtain a high-quality genome of a specific bacterium.

One of the globally monitored marine bacterial lineages, which includes strains associated with human diseases and connected with mass mortality events of economically and ecologically important marine organisms, is the genus *Vibrio* [14–17]. This genetically diverse lineage is part of the ambient microbiome in estuaries, coastal seawater, deep sea, and even marine sediments [17, 18]. Although *Vibrio* spp. usually comprises a minor fraction of the bacterial community (< 1%) [19, 20], it can become abundant under specific environmental conditions [21, 22]. For example, the increase in abundance of *Vibrio* spp. was related to the rise of seawater temperature and the decrease in seawater salinity [20]. Higher seawater temperatures were also associated with higher expression of its virulence genes in *Vibrio harveyi* [23]. This relationship is important in the context of projected future changes of coastal habitats (e.g., increase of seawater temperatures, droughts, sea level rise) [17, 24–27]. However, due to high genomic and phenotypic similarity, conventional analyses relying on marker genes or phenotypes frequently encounter challenges in distinguishing between closely related pathogenic and non-pathogenic *Vibrio* lineages [28–30], making it challenging to monitor and control *Vibrio*-associated disease outbreaks [31, 32]. In addition, as *Vibrio*-associated infections have become more frequent in recent years [25], it is crucial to improve our understanding of the functional and ecological traits of this bacterial lineage.

Previous microbial monitoring, performed by diversity analysis using 16S rRNA gene amplicon sequencing, revealed that *Vibrio* spp. are members of the core ambient microbiome of the coastal ecosystem in the northern Adriatic Sea [33–35], specifically in the shallow, semi-enclosed Gulf of Trieste, characterized by high salinity and temperature fluctuations. However, the resolution of these analyses was too low to accurately determine the taxonomy of the detected *Vibrio* spp. and to determine whether they are pathogenic. Therefore, our objective was to perform WGS of *Vibrio* spp. isolates from coastal waters of the Northern Adriatic Sea to acquire their accurate taxonomic identification and to elucidate their functional and pathogenic potential. Genomic analysis of two selected isolates revealed a cross-contamination event between them, where one *Vibrio* isolate was introduced into the culture of the second isolate during laboratory processing. Having sequencing libraries from both an axenic and non-axenic culture of the same *Vibrio* isolate allowed us to test the potential for recovering similar high-quality genomes from both cultures. We report here the result of our thorough bioinformatic analysis, which we believe will be useful to our peers dealing with this common analytical challenge.

## Results and discussion

### Sequencing and genome assembly

To identify *Vibrio* candidates for WGS, we carried out taxonomic classification of a collection of bacterial isolates from the Gulf of Trieste using Sanger sequencing of ~ 1400 bp of 16S rRNA gene (27F – 1492R). The two selected isolates were affiliated with the *Vibrionaceae* family (Table 1). However, the 16S rRNA gene did not allow accurate classification at a lower taxonomic rank (e.g., genus), a common problem with marker gene-based analyses of *Vibrio* lineages [31, 32].

**Table 1:** Bacterial cultures analyzed in this study and their closest relatives according to 16S rRNA

Internal Code^a^	Isolation source	Isolation date	5 closest relatives in GeneBank; Accession number	Percent identity	Max Score	Query cover
BF5_0283	Surface seawater (5 m depth) at offshore station (45°32′55.50″N, 13°33′2.52″E)	15/07/2010	*Vibrio rotiferianus* strain KP40.3; MT020420.1	*98,90%*	*1293*	*100%*
			*V. parahaemolyticus* strain MAI-4; MN316590.1	*98,90%*	*1293*	*100%*
			*V. campbellii* strain DS1907-1YC1–1, MT269634.1	*98,90%*	*1291*	*100%*
			*V. harveyi* strain XSH1, MT071600.1	*98,90%*	*1291*	*100%*
			*V. campbellii* strain 3–35; MN938230.1	*98,90%*	*1291*	*100%*
Mt009	Bottom seawater (8 m depth) at wastewater treatment plant discharge (45°33′31.00″N, 13°44′37.30″E)	04/12/2018	*Vibrio* sp. strain LMG 19840; AJ316207.1	*99,77%*	*2444*	*100%*
			*Enterovibrio* sp. S20CA; KF188499.1	*99,77%*	*2444*	*100%*
			*Enterovibrio norvegicus* strain LMG 19839; NR_042082.1	*99,62%*	*2436*	*100%*
			*Vibrio* sp. EN276; AB038023.1	*99,62%*	*2431*	*100%*
			*Enterovibrio* sp. AU32B; LN878395.1	*99,03%*	*2388*	*100%*

^**a**^ 16S rRNA amplicon obtained by Sanger sequencing; BF5_0283 Accession No. JX864957, for Mt009 see Additional file 3

**Fig. 1:**
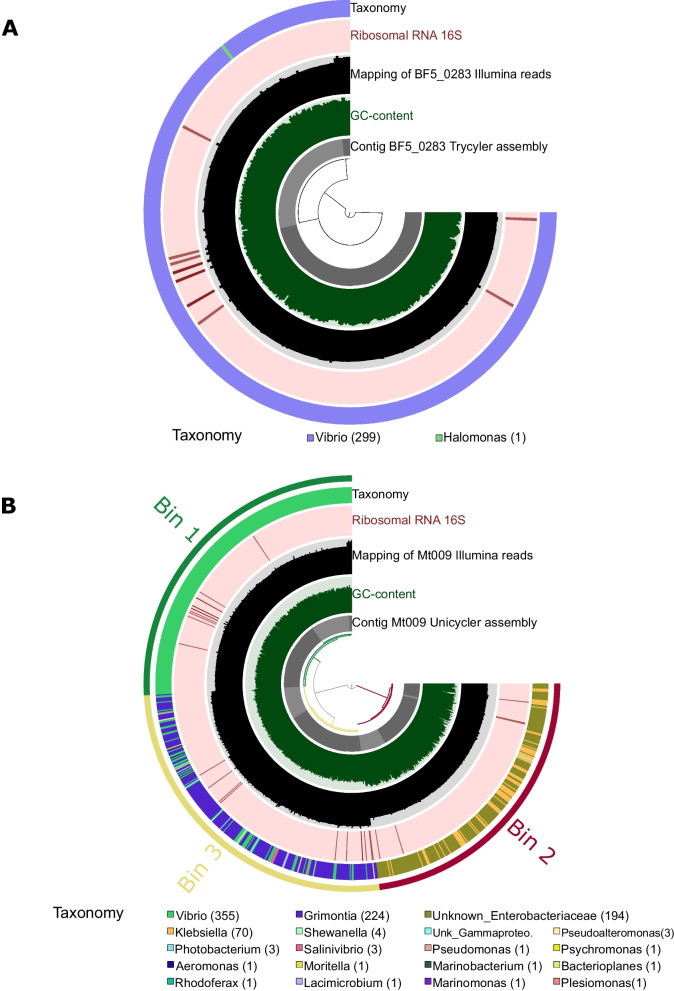
Refinement of the assembled genomes. Graphical representation of the BF5_0283 Trycycler assembly (**a**) and the Mt009 Unicycler assembly (**b**) along with associated data: GC-content, mapping of Illumina reads, 16S rRNA and genes taxonomy. The bins in (**b**) were manually refined based on differences in mean coverage (mapping of Illumina short reads), differences in GC content and gene taxonomy

Genomic DNA from cultures of both isolates was sequenced in parallel using long (MinION, Oxford Nanopore Technology) and short-read (Illumina) techniques (Table 2). To assemble bacterial genomes, we implemented the Trycycler workflow, which produces a consensus assembly based on manually selected contig clusters from multiple long-read-only assemblers (methodology described elsewhere [36]). In our case we combined genome assemblies from three different assembly tools (Flye [37], Miniasm+Minipolish [38, 39] and Raven [40]), followed by post-assembly long- and short-read polishing (described in detail in Methods). Genomic sequences assembled from the BF5_0283 culture formed three distinct contig clusters that resulted in a consensus sequence of three circular DNA molecules with a total length of 6.03 Mb (Additional file 2: Fig. S1 A, Fig. 1a). In contrast, a similar approach on sequences from the Mt009 culture did not produce clear clusters (Additional file 2: Fig. S1 B) and the resulting three consensus DNA sequences had a total length of 7.66 Mb. In an attempt to improve the genomic assembly from the Mt009 culture, we implemented two other approaches: (1) short read-first hybrid assembly tool Unicycler - specifically designed for the assembly of bacterial genomes [41] and (2) long-read metagenome assembler metaFlye [42]. Both tools, the Unicycler and the metaFlye, resulted in even larger assemblies (17.48 and 19.92 Mb, respectively) and a higher number of contigs (21 and 188, respectively), compared to Trycycler (Table 2). The Trycycler consensus contigs of both cultures, as well as the contigs in other Mt009 assembly attempts covered approximately 71% of the *V. campbellii* ATCC BAA 1116 genome (Table 2, metaQUAST calculation). However, the assembly from the Mt009 culture also covered a large fraction of *Enterovibrio norvegicus* Alg239-V16 and *Klebsiella pneumoniae* KCTC 2242 genomes, indicating that Mt009 culture was either non-axenic or contaminated.

**Table 2:** Sequencing information and assembly statistics for BF5_0283 Trycycler and Mt009 Trycycler, Unicycler and metaFlye assembly

	**BF5_0283**	**Mt009**		
**Sequencing information**				
**ONT library size** ^**a**^	1,022,088	685,827		
**ONT mean read length [b]** ^**a**^	1887.3	1910.9		
**Illumina library size (pair-end)**	2 × 27,521,418	2 × 23,515,083		
**Illumina mean read length [b]**	75	75		
**Assembly method**	**Trycycler**	**Trycycler**	**Unicycler**	**metaFlye**
**Description**	Long-read assembly (followed by long and short-read polishing)	Long-read assembly (followed by long and short-read polishing)	Short-read-first hybrid assembly (short read assembly, followed by long-read plus contig assembly) and polishing	Long read assembly
**Total length of assembly (bp)**	6,034,252	7,658,097	17,479,769	19,919,184
**Total number of contigs in assembly**	3	3	21	188
**Number of contigs in assembly (> = 50,000 bp)**	3	3	9	17
**Average depth of ONT sequencing coverage** ^**a**^	319.7x	*171.1x*	75.0x	65.8x
**Average depth of Illumina sequencing coverage**	342.1x	*230.3x*	100.1x	88.6x
**Reference coverage (%)**^**b**^				
***Vibrio campbellii ATCC BAA 1116***	71.49%	71.41%	71.55%	71.55%
***Enterovibrio norvegicus Alg239-V16***	0.04%	24.07%	82.87%	82.90%
***Klebsiella pneumoniae KCTC 2242***	0.01%	0.01%	93.39%	90.61%

^**a**^ ONT (Oxford Nanopore Technology)

^**b**^ Reference coverage is the alignment between reference strain and assembled strains computed with metaQUAST 5.0.2

### Tracking the contamination

To investigate whether the contamination of Mt009 occurred already during isolation, we used dedicated polymerase chain reaction (PCR) primers (Vca-hly-5 / Vca-hly-3 and KP878-F / KP878-R) to test for the presence of *V. campbellii* and *K. pneumoniae* in the cryo-preserved stock of the initial Mt009 and BF_0283 isolate. The PCR results did not confirm the presence of *V. campbellii* in the initial cryo-preserved culture stock of Mt009 but did show a weak signal of *K. pneumonia* (Additional file 2: Fig. S3 A, Fig. S3 B). The presence of *E. norvegicus* was not tested, due to the lack of published taxa-specific PCR primers. Contamination may have also occurred during the sequencing process (i.e., cross-barcode contamination). However, usually in such cases the contaminated contigs show lower than expected read depth [43], which was not the case in Mt009, as revealed by our further analysis (Fig. 1b, Additional file 1: Table S1). Taken together, these results suggested that the initial Mt009 isolate most likely contained a co-culture of *K. pneumoniae* and *E. norvegicus*, while *V. campbellii* was introduced in the laboratory during secondary cultivation.

## Retrieving the *Vibrio campbellii* genome from the non-axenic culture

To retrieve the genome of interest from the non-axenic culture, we addressed the Mt009 sequencing dataset as a metagenome and performed binning of the assembled contigs. Through a combination of Illumina short-read coverage and G + C content, we were able to manually refine three genomic bins from the Mt009 assembly (Fig. 1b, Additional file 1: Table S1). Based on single copy gene taxonomy, as well as BLASTn search of the 16S rRNA genes, the bins were assigned to *V. campbellii* (Mt009_b1), *E. norvegicus* (Mt009_b2), and *K. pneumoniae* (Mt009_b3) (Additional file 1: Table S2). Unicycler has been previously suggested to retrieve metagenome assembled genomes (MAGs) from metagenomics samples with a combination of short- and long-reads [44]. Indeed, out of the three tested tools, the binned contigs assembled using Unicycler gave the most complete genome and were therefore chosen as the consensus for further genomic analyses of the Mt009 dataset (Additional file 2: Fig. S2).

### Comparison of assembled genomes from axenic and non-axenic cultures

Our WGS study resulted in two *V. campbellii* genomes, the first assembled from the axenic culture (BF5_0283) and the second acquired from a non-axenic culture (Mt009). In accordance with the known structure of the *V. campbellii* genome, both assembled genomes had two circular chromosomes of 3.7 and 2.1 Mbp (Table 3). The number and length of plasmids varies between different *V. campbellii* strains [45, 46], and in the case of the assembled genomes both likely contain a putative plasmid of 150 Kbp (Table 3). The particularly high Average Nucleotide Identity (ANI) of 99.98% between the assembled *V. campbellii* genomes (Additional file 1: Table S6) strongly indicates that most likely there was a cross-contamination event between the two cultures and that we generated the genome of the same *V. campbellii* strain (BF5_0283), once from an axenic culture and once “salvaged” from a contaminated one.

**Table 3:** Comparison of genomic features between BF5_0283 and Mt009_b1 assemblies

	Value		Difference between assemblies
Feature	BF5_0283	Mt009_b1	BF5 - Mt009_b1
Chr I size (bp) ^a^	3,734,099	3,729,301	**4798**
Chr II size (bp) ^a^	2,149,670	2,149,712	**−42**
P size (bp) ^a^	150,483	150,486	**−3**
Chr I G + C content ^a^	45.34	45.34	**0**
Chr II G + C content ^a^	45.06	45.06	**0**
P I G + C content	45.01	45.01	**0**
Protein-coding genes	5628	5571	**57**
rRNAs	24	24	**0**

^**a**^ Chr I, Chr II and P stands for chromosome I, chromosome II and plasmid

The unexpected cross-contamination allowed us to compare the two assemblies (BF5_0283 and Mt009_b1) to assess the extent of genomic information loss when performing WGS from a non-axenic culture. BLASTn was used for bidirectional best hit analysis (i.e., identification of the pairs of genes in two different genomes that are more similar to each other than to any other gene). We found that 5394 genes (the vast majority of the genes) were similarly represented in both assemblies (Additional file 1: Table 3, Table 4). A total of 24 genes from the BF5_0283 assembly, mostly with unknown functions, was missing in the Mt009_b1 assembly (Additional file 1: Table 3). However, there were 50 genes in the Mt009_b1 assembly not present in the BF5_0283 (Additional file 1: Table 4). The mean coverage of these 50 additional genes was slightly higher than the mean coverage of all genes in the Mt009_b1 assembly (86.31 vs. 83.46, respectively), potentially suggesting that they could be an artifact introduced from the other binned genomes in the non-axenic culture. Nonetheless, this comparison confirmed that we successfully assembled an almost identical genome of a *V. campbellii* isolated from both, the axenic and contaminated culture.

### Genomic comparison to other *V. campbellii* isolates

**Fig. 2:**
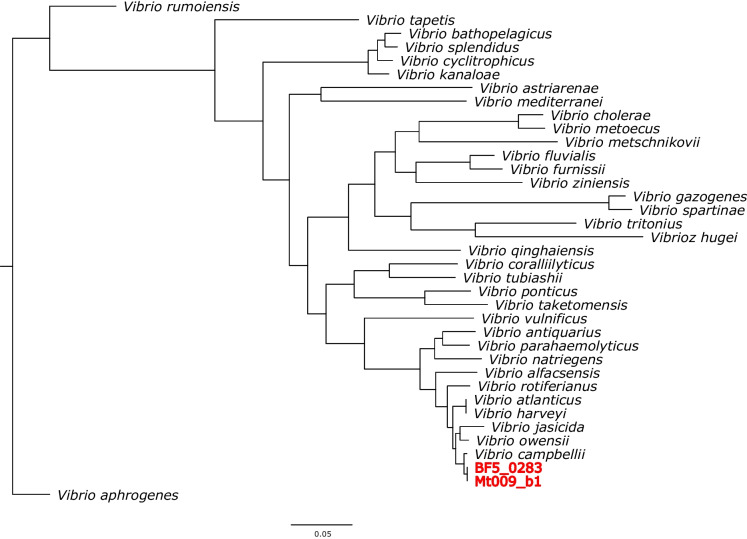
Phylogeny of *Vibrio* genomes based on single-copy core orthologues. An approximately-maximum-likelihood phylogenetic tree representing 32 *Vibrio* genomes from NCBI (Additional file 1: Supplementary Table S5) and two genomes assembled in this study (BF5_0283 and Mt009_b1)

To confirm the taxonomic affiliation of assembled genome, we collected all currently available complete representative genomes of *Vibrio* spp. from NCBI (National Center for Biotechnology Information). In total, 32 representative genomes were collected, and three additional complete genomes representing *Vibrio* species commonly found in coastal marine environments (*Vibrio coralliilyticus*, *V. mediterranei, V. splendidus*) were added (Additional file 1: Table S5). The phylogenetic tree, constructed based on concatenated alignment of 1027 single copy amino acid sequences of orthologous genes, showed that both assemblies are consistently affiliated with *V. campbellii* (Fig. 2). Further analysis of the BF5_0283 and Mt009_b1 assemblies and 10 complete *V. campbellii* genomes from NCBI (Additional file 1: Table S7) revealed that the isolated strain clustered with 6 *V. campbellii* strains in Group 1. In accordance with previous studies, *V. cambpellii* contains two clusters, Group 1 - isolates originating from aquatic animals and biofilms [47, 48], and Group 2 – represents isolates of oceanic origin [49, 50].

**Fig. 3:**
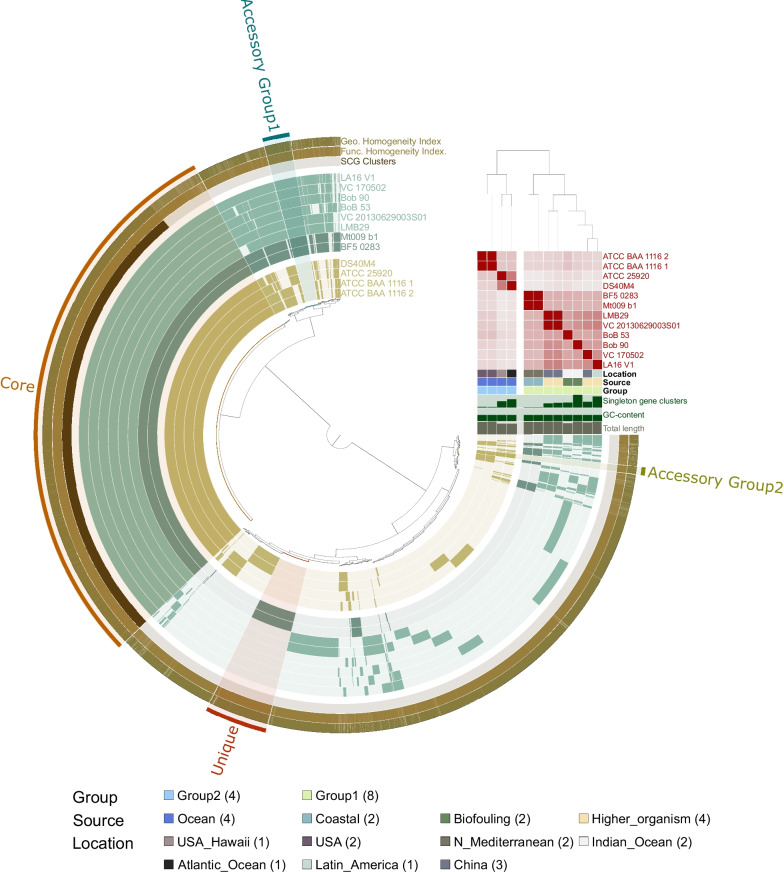
*Vibrio campbellii* pangenome. The radial layers present genomes ordered according to their phylogenetic relationship based on maximum likelihood phylogenetic tree constructed from single copy orthologous genes. ANIb values are shown as heatmap with high similarity in red and lower similarity in gray color. The dark and light color of bars in radial layers show presence and absence, respectively, of 9318 gene clusters. Gene clusters (GCs) are organized based on their distribution across genomes using Euclidean distance and Ward ordination (gene tree in the center of pangenome). The “Core” collection corresponds to GCs containing genes from all genomes. The “Accessory Group 1” and “Accessory Group 2” refers to genes characteristic to Group 1 and Group 2. The “Unique” collection refers to genes unique for the new assembled genome in this study (BF5_0283 and Mt009_b1). Collections are marked in the outermost radial layer. Colored squares below the ANIb value heatmap provide additional data for each isolate

**Fig. 4:**
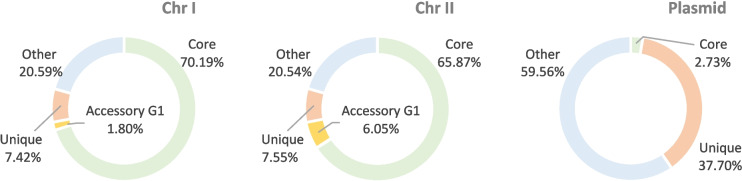
Proportion of core genome, accessory Group 1 (G1) and Unique GCs in the chromosomes and the plasmid of BF5_0283

Pangenome analysis performed with both, BF5_0283 and Mt009_b1 assemblies and 10 reference genomes of *V. campbellii* resulted in 9318 functional gene clusters (GCs) (Fig. 3). The GCs could be divided into three collections: ‘core genome’ – GCs shared among all strains (39.0% of all GCs), ‘accessory’ - GCs specific to a subset of the genomes clustering into Group 1 and Group 2 (1.9 and 0.6% of all GCs for Group 1 and Group 2, respectively), as well as ‘unique’ - GCs found in individual strains (4% of all GCs for BF5_0283). The ‘core genome’ contained the majority of chromosomal genes of BF5_0283 (~ 70 and 66% of genes on Chr I and Chr II, respectively), indicating their high conservation among *V. campbellii* (Fig. 4). The core genome of *V. campbellii* comprised a set of conserved genomic functions, with the most abundant COG categories being signal transduction mechanisms (8% of core genome GCs) and amino acid transport and metabolism (7.6%) (Additional file 2: Fig. S4, Fig. S5 A), which suggests involvement of this lineage in protein turnover in the seawater. The accessory GCs of Group 1, to which our isolate belongs, contained mainly genes connected with intracellular trafficking, secretion, and vesicular transport (14.1% of accessory GCs in Group 1), as well as signal transduction mechanisms (9.1%) (Additional file 2: Fig. S5 B), which may imply the specialization of these strains for intercellular interactions (e.g., with their host). On the KOfam level, we found type VI secretion systems (T6SS) (e.g., K11902, K11899, K11898), accessory colonization factors *acfA* and *acfD* (i.e., K10939, K10936), toxin-antitoxin systems genes *ccdA* and *ccdB* (K19163, K19164), and the toxin gene *hipA* (K07154) associated with Group 1 (Additional file 1: Table S9). All these markers are involved in the pathogenesis of *Vibrio* spp. [30, 51, 52]. It was previously reported that T6SS and HipA might contribute fitness advantages to the AHPND-causing *V. parahaemolyticus* over competing bacteria and in this way facilitating shrimp infection [53, 54]. T6SS systems are complex systems that inject so-called ‘anti-bacterial’ and ‘anti-eukaryotic’ effector proteins into target cells, targeting both, eukaryotic hosts and bacterial competitors [55, 56], while the serine/threonine protein kinase HipA is a toxin that causes inhibition of cell growth [57]. In contrast to previous reports, our results did not reveal functions related to antibiotic transport and galactose metabolic process associated with Group 1 [29]. The accessory GCs collection for Group 2 mainly contained genes related to transcription (8.1% of the genes in the accessory collection of Group 2), inorganic ion transport and metabolism (6.5%), and general function (6.1%), representing the three most abundant COG categories (Additional file 2: Fig. S5 B). On the KOfam level, we found sensory rhodopsin (i.e., K04643) (Additional file 1: Table S9), which suggests mixotrophy of Group 2. Although the presence of this gene has been previously described in *V. campbellii* BAA-1116 [58], we found that it is specific to all genomes in Group 2. We hypothesize that since these isolates originate from ocean waters, they probably undergo adaptation to survive in nutrient-poor environments. Interestingly, ‘unique’ GCs of our isolate accounted for ca. 40% of all genes present on its putative plasmid, with only a small portion of the plasmid-associated genes being part of the *V. campbellii* core genome and none associated with accessory genes of the Group 1 cluster (Fig. 4).

## Exploring the plasmid of the novel *Vibrio campbellii* genome

**Fig. 5:**
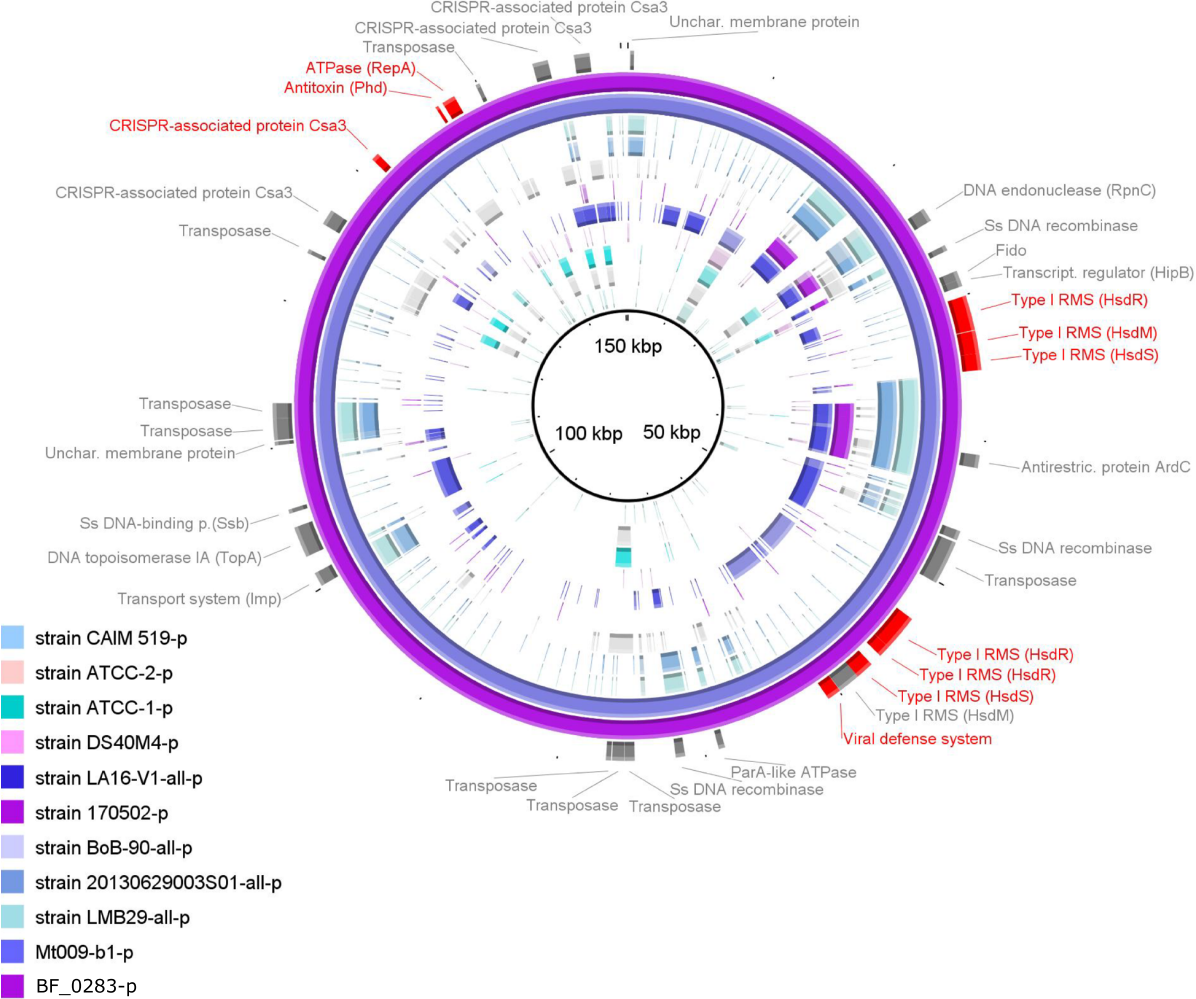
BRIG visualization of comparative sequence analysis of the plasmid. BLASTn was used to align plasmid sequences from newly assembled genomes BF5_0283 and Mt009_b1 with all *V. campbellii* plasmids in PLSDB. Circles from outside to inside are plasmid sequences of BF5_0283, Mt009_b1, LMB29, 20130629003S01, BoB-90, 170,502, LA16-V1, DS40M4, ATCC-1, ATCC-2, CAIM 519. In cases where the genome contained more plasmids, all plasmids were aligned. The intensity in colors indicates 100% (higher intensity), 70% (medium intensity) and 50% (low intensity) identity. The outermost circle shows protein coding genes with hits in *V. campbellii* plasmids from PLSDB (gray), and selected unique genes of newly assembled plasmids (red). Complete annotation of common genes is available in Additional file 1: Table S12

We compared the sequence of the identified putative plasmid of BF5_0283 to previously characterized plasmids in the plasmid database PLSDB (v. 2021_06_23_v2) (max_pvalue 0.1, max_distance 0.2) [59]. According to Mash distances, the most closely related plasmids were found in *V. campbellii* strains: plasmid pLA16–1 in strain LA16-V1 (Mash distance 0.1168), plasmid pLMB143 in strain LMB29 (0.1354), plasmid pVCGX3 in strain 20130629003S01 (0.1354), and plasmid pLA16–4 in strain LA16-V1 (0.1370) (Additional file 1: Table S11). More distant plasmids were found in *V. parahaemolyticus*, *V. owensii*, and other *V. campbellii* genomes. The majority of related plasmids were isolated from the host organism *Penaeus vannamei* (52%), and some were isolated from AHPND infected shrimps (23%) (Additional file 1: Table S11) [47, 48, 60]. Although only parts of the putative plasmid were similar to other *V. campbellii* plasmids, these shared genes such as the anti-restriction protein gene *ardC* and CRISPR Csa3 system (Fig. 5). The presence of CRISPR Csa3 system suggests that these plasmids could provide a defense function, since this system is involved in protecting the cell against foreign DNA, such as bacteriophages [61–64].

The unique fraction of the putative plasmid of BF5_0283 comprised two complete sets of genes of the Type I restriction-modification system (Fig. 5). This is surprising, since previous studies reported that many individual genes involved in the Type I R-M system are usually present on plasmids, but only few complete systems [65]. The Type I R-M system consists of genes for methyltransferase (*hsdM*) that specifically methylate DNA, restriction endonuclease (*hsdR*) cleaving DNA that has not been properly modified (i.e., methylated), and genes for specificity (*hsdS*) determining the recognition sequence of restriction and modification activities [66]. The presence of this system has been previously connected with a ‘selfish-behavior’ of the plasmid carrying the R-M gene complex, since the loss of the R-M gene complex can lead to cell death, because the balance of methyltransferases and restriction endonucleases in a cell is disturbed [67, 68]. This suggests that plasmid containing R-M genes cannot be eliminated from the cell or displaced by the plasmid lacking this gene complex.

Interestingly, the *ardC* genes observed in the putative plasmid of BF5_0283 have anti-restriction activity against the type I R-M system, which enables the plasmid to overpass the R-M systems of the recipient cell once they are transferred by conjugation [69]. In that way, plasmids broaden their host range [70]. This, together with numerous transposases playing a role in horizontal gene transfer [71] suggests that there is potential for propagation of plasmid genes in coastal systems, as it was previously shown for *Vibrio* spp. [5, 72, 73].

## Conclusions

Our study highlights the power of whole genome sequencing for accurate taxonomic identification and unraveling the pathogenic potential of emerging environmental pathogens. In fact, our analysis revealed that the genome of *Vibrio campbellii* isolated from the northern Adriatic Sea carries genes for T6SS type VI secretion systems, known for their role in pathogenesis and interbacterial antagonism, as well as novel putative *Vibrio* plasmid, both of which should be further explored. Besides, our approach to salvage a high-quality genome of the bacteria from a contaminated culture using state-of-the-art bioinformatics tools and a sufficient sequencing effort can be implemented when dealing with common issues of non-axenic cultures. This approach can be also applied, for example, to study bacteria that exhibit co-culture dependence (e.g., Prochlorococcus) [14, 15] or to study interspecific interactions [16] or to reduce the time and costs of analyses, such as proposed for genomic epidemiology studies [17]. Last but not least, high quality genome sequences can also serve as baseline for the development of new monitoring approaches (e.g., more specific primers for more reliable monitoring than with the 16S rRNA approach), which will allow us to track and control propagation of emerging pathogens in marine coastal ecosystems. This is crucial to constrain disease outbreaks, which will help maintaining ecosystem services in the future.

## Methods

### Isolation, culture condition and DNA sequencing

For bacterial isolation, a defined volume of seawater was spread on modified ZoBell solid agar media [74] and incubated in the dark at 21 °C by gently agitating for 48 h. Single colonies were clean streaked once and inoculated into ZoBell liquid medium and incubated in the dark at 21 °C for 24 h. Bacterial genomic DNA for 16S rRNA Sanger sequencing was extracted immediately, with a modified Chelex-based procedure [75], amplified with universal primers 27F and 1492R, and sent for Sanger sequencing at Macrogen Inc. (Accession number JX864957 and Additional file 3). Both isolates were stored at the culture collection of the Marine Biology Station Piran, Slovenia (in 30% glycerol at − 80 °C).

Each isolate from the cryo-preserved stock was re-grown on ZoBell agar plates (at 24 °C for 72 h in the dark). A single colony was picked from the agar plate, inoculated into 6 mL of ZoBell liquid medium, and incubated at room temperature in the dark on a shaker. For each isolate, four 1 mL replicates of the liquid culture were pelleted by centrifugation at 4000x *g* for 3 min. The bacterial pellets were then shipped on dry ice to the sequencing facility (Microsynth AG, Balgach, Switzerland) where high molecular weight DNA was extracted. The DNA was sequenced using the long-read MinION ONT (Oxford Nanopore Technologies, Oxford, United Kingdom) technique and complemented by short-read paired-end (2 × 75 bp) sequencing on Illumina NextSeq (Illumina, San Diego, CA, USA).

### Contamination check using polymerase chain reaction

Cryo-preserved stocks were re-grown using the same culturing conditions as described above. Bacterial genomic DNA was isolated with a modified Chelex-based procedure [75] and amplified by PCR reaction using universal 16S rRNA bacterial primers (27F and 1492R) or species-specific primers (Vca-hly-5 / Vca-hly-3 targeting haemolysin (*hly*) gene of *V. campbellii* and KP878-F / KP878-R targeting transferase gene of *K. pneumoniae*) (Table 4). A total of 50 μL of PCR mixture was prepared for each isolate and each primer pair with a suitable primer concentration (0.5 μM for universal primers, 0.25 μM for species-specific primers), 1X Tris KCl-MgCl_2_, 1.5 mM MgCl_2_, 0.2 mM dNTP and 0.3 U Taq polymerase. The PCR protocol was as follows: 5 min of initial denaturation at 94 °C, 30 cycles of 30 sec denaturation at 94 °C, 30 sec of primer annealing at 54 °C, 30 sec for extension at 72 °C, followed by a final extension for 5 min at 72 °C.

**Table 4:** Selection of species-specific primers for PCR reaction

Taxa	Primer	Reference	Blast against BIN collection (number of identities)
*V. campbellii*	**Vca-hly-5: CTATTGGTGGAACGCAC (17)**	[30, 76]	***V. campbellii*** **(BIN 1):** F: 17/17 R: 19/19 ***E. norvegicus*** **(BIN 2):** F: 14/14 R: 13/13 ***K. pneumoniae*** **(BIN 3):** F: 16/17 R: 13/19
	**Vca-hly-3: GTATTCTGTCCATACAAAC (19)**		
*K. pneumoniae*	**KP878-F: ACCGATAACCAGCCTGACTT (20)**	[77]	***V. campbellii*** **(BIN 1):** F: 14/20 R: 13/20 ***E. norvegicus*** **(BIN 2):** F: 14/20 R: 13/20 ***K. pneumoniae*** **(BIN 3):** F: 20/20 R: 20/20
	**KP878-R: CTTTCTTCTGCCCACTGTTG (20)**		

### Genome assembly

Raw reads were quality-filtered using the Filtlong tool for long reads (keep percent 75%) [78] and fastp (default thresholds) [79] for short reads. Assembly of isolate BF5_0283 was performed using the Trycycler tool [36] combining multiple separate long-read assemblies of the same genome. Assemblies were created by subsampling 12 long-read sets assembled using the assembling tool Flye [37], Miniasm+Minipolish [38, 39] and Raven [40]. Trycycler contigs tree was visualized using iTOL (v 6.8.1) [80]. Long-read polishing of the consensus long-read assembly was done with Medaka (v. 1.4.4) [81] and short-read polishing with Pilon tool (v. 1.24) [82].

Mt009 was assembled using three different methods. First, Trycycler was used for long-read assembly followed by long- and short-read polishing as described for BF5_0283. Second, the genome was assembled using Unicycler short-read-first hybrid assembly tool [41] which uses SPAdes for short read-assembly [83], followed with Miniasm long-read plus contig assembly and Racon polishing [84]. Third, metaFlye [42] was used for long-read assembly. Sequences shorter than 10 Kb were removed. Quality assessment of all assemblies was done with metaQUAST tool (v. 5.0.2) [85] without providing reference genomes.

### Genome annotation and refinement

The assembled genomes were first annotated using Anvi’o v. 7 [86]. Briefly, for Anvi’o annotation we used ‘anvi-gen-contigs-database’ to construct the contig database for each assembly, which uses Prodigal [87] to identify ORFs in each contig. We ran HMM (Hidden Markov models) with ‘anvi-run-hmms’ and assigned functions to the genes by alignment against the COG database [88, 89] with the ‘anvi-run-ncbi-cogs’ program. We also used ‘anvi-run-kegg-kofams’, which uses hmmsearch to find hits from KOfam, database of KEGG Orthologs (KOs) [90]. Gene taxonomy was annotated with kaiju classifier [91] and ‘anvio-run-scg-taxonomy’. Short-read mapping to the assembled genome was done using bowtie2 [92]. An anvi’o profile database was generated storing coverage statistics using ‘anvi-profile’ with ‘--cluster-contigs’ option. We manually refined the bins in the Mt009 assembly to identify bacterial genomes in this sample within the ‘anvi-interactive’ interface. The taxonomy of each bin was assigned by exporting and alignment all 16S genes and by inspecting the taxonomy of single-copy genes with ‘anvi-summarize’. We used ‘anvi-split’ to split the Mt009 sample into three separated genomes (Mt009_b1, Mt009_b2, Mt009_b3).

### Comparative genomics analysis

For comparative functional analyses of the *V. campbellii* genomes assembled in this study (BF5_0283 and Mt009_b1) and the reference *Vibrio* spp. genomes, we annotated the assemblies on the RAST Server [93]. This was done by importing fasta files into the web-based annotation service, running annotation (RASTtk annotation scheme). To compare BF5_0283 and Mt009_b1 assemblies, Bidirectional Best Hits (BBH) were calculated in Seed Viewer [94]. The exported annotated genomes in GeneBank format were imported into Anvi’o with ‘anvi-script-process-genbank’ and a contig database was created using ‘anvi-gen-contigs-database’ with ‘--external-gene-calls’ flag. The annotation was completed with the COG and KOfam database as described above.

*Vibrio* spp. genomes were downloaded from NCBI and annotated (with RAST tool and Anvi’o) as described above. To construct the phylogenetic tree based on orthologous genes, we extracted and aligned genes from single-copy gene clusters present in all 37 genomes with ‘anvi-get-sequences-for-gene-clusters’ program. Nucleotide positions missing in more than 50% of sequences were removed (with ‘trimal’). The amino acid translated phylogenetic tree was constructed with IQ-TREE (v. 2.0.3) (options -m WAG, −bb 1000, to specify WAG substitution model and the number of bootstrap replicates to 1000 – recommended values) [95]. The resulting phylogeny was subsequently rooted and edited in FigTree (v 1.4.4) [96]. To explore similarities across genomes of *Vibrio* species, the average nucleotide identity (ANI) value was calculated with ‘anvi-compute-genome-similarity’ using Phyton module PyANI [97].

The pangenome was created to compare genomes assembled in this study with 10 complete genomes of *V. campbellii* retrieved from NCBI. FASTA files of the public genomes were downloaded and processed and annotated as described for BF5_0283 and Mt009_b1 (using RAST and Anvi’o). The pangenome was constructed following the pangenomics workflow in Anvi’o v. 7.1 [98]. Briefly, ‘anvi-gen-genomes-storage’ was used to create the genome database and the ‘anvi-pan-genome’ program that uses BLASTp for amino acid sequence similarity search, and the MCL algorithm to identify gene clusters in the amino acid sequence similarity results [99]. The inflation parameter was set to 10 to increase the sensitivity of the algorithm, suggested for closely related genomes [99]. ANI was calculated with ‘anvi-compute-ani’ using the PyANI program. Genomes in the *V. campbellii* pangenome were organized based on the single-copy core genes tree, constructed with IQ-TREE [95]. Gene clusters were grouped into core bin containing gene clusters present in all genomes, accessory bins with gene clusters present in genomes belonging to a specific group and unique bins with gene clusters specific to the genomes assembled in this study. Data were exported with ‘anvi-summarize’. Heatmaps of genes with COG annotations in different collections, and barplots of genes with COG annotations on chromosomes and the plasmid were plotted in R [100] using ‘tidyr’ [101], ‘dplyr’ [102], ‘ggplot2’ [103] and ‘forcats’ [104] packages.

We identified functions enriched in *V. campbellii* Group 1 or Group 2 in our pangenome with the program ‘anvi-compute-functional-enrichment-in-pan’. The program calculates functional enrichment scores using the Rao score test for equality of proportions. False detection rate correction is applied to the *p*-values to account for multiple tests.

### Plasmid exploration and gene map visualization

For plasmid exploration, the sequence of the plasmid from the BF5_0283 genome was used. The similarity comparison of the novel assembled plasmid with reference plasmids was done by Mash distance search in publicly available plasmid sequences (PLSDB) [59]. The distance ranges from 0 (identical) to 1 (highly unrelated). We limited the search with a maximum *p*-value of 0.1 and a maximum distance of 0.2. To explore which reference plasmids contain genes similar to our plasmid, we extracted gene sequences with ‘anvi-get-sequences-for-gene-calls’ and searched with BLASTn search in PLSDB with the default parameters: minimal identity 80% and minimal query coverage/HSP 90%. Nucleotide alignment and visualization of the plasmid assembled in this study and PLSDB were performed using BRIG v 0.95 [105]. All final figures were edited using the vector graphics editor Inkscape v 1.1 [106].

### Supplementary Information

**Additional file 1: Table S1**. A summary of mean coverage of mapped reads. **Table S2**. A summary of three bins in sample Mt009. **Table S3**. RBH for BF5_0283 reference genome. **Table S4**. RBH for Mt009_b1 reference genome. **Table S5**. List of Vibrio spp. genomes. **Table S6**. ANI values between Vibrio spp. genomes. **Table S7**. Vibrio campbellii comparison. **Table S8**. ANI values between Vibrio campbellii genomes. **Table S9**. Enriched KOfam domains. **Table S10**. COG functions present only on plasmid on new genomes. **Table S11**. Similar plasmids in PLSDB. **Table S12**. Shared and unique plasmid genes. **Table S13**. Strain database.

**Additional file 2: Figure S1**. Trycycler contigs tree. **Figure S2**. Graphical presentation of contigs from Mt009 assemblies along with associated data with “anvi-interactive” function. **Figure S3**. Results of PCR reaction with taxa-specific primers. **Figure S4**. Number of genes assigned to the COG category on chromosomes (ChrI, ChrII) and the plasmid (P). **Figure S5**. Gene abundance heat map, representing abundance of genes in *V. campbelli* genomes, belonging to different COG categories.

**Additional file 3:** This is fasta file of 16S rRNA Sanger sequence of Mt009 sample.

## Data Availability

The datasets supporting the conclusions of this article are available in the National Centre for Biotechnology Information (NCBI) database (https://www.ncbi.nlm.nih.gov/) under project accession number PRJEB58817. The raw Oxford Nanopore and Illumina NovaSeq reads were deposited under accession numbers ERR10772267, ERR10762505 (BF5_0283) and ERR10777132, ERR10777120 (Mt009). Assembled genomes are deposited under accessions GCA_948151475.1 (BF5_0283) and GCA_948331105.1 (Mt009_b1). 16S rRNA Sanger sequence is deposited in the GenBank (NCBI) under accession JX864957 (BF5_0283) or included in Additional file 3 (Mt009). All other genome sequences analyzed in the current study are available from the NCBI database and the accession numbers are listed in the Additional file 1: Supplementary Table S5 and Supplementary Table S7.
