# Supplementary material for: Recovering high-quality bacterial genomes from cross-contaminated cultures: a case study of marine Vibrio campbellii
Source: BMC Genomics. 2024 Feb 6;25:146. doi: 10.1186/s12864-024-10062-2 (PMC10845552; doi:10.1186/s12864-024-10062-2)
Supplement: Supplementary file 2 — Additional file 2: Figure S1. Trycycler contigs tree. Figure S2. Graphical presentation of contigs from Mt009 assemblies along with associated data with “anvi-interactive” function. Figure S3. Results of PCR reaction with taxa-specific primers. Figure S4. Number of genes assigned to the COG category on chromosomes (ChrI, ChrII) and the plasmid (P). Figure S5. Gene abundance heat map, representing abundance of genes in V. campbelli genomes, belonging to different COG categories. [file 12864_2024_10062_MOESM2_ESM.pdf]

Figure S1: Trycycler contigs tree. Trycycler tool requires manual intervention; therefore, its output is not deterministic. In the first step of Trycycler workflow, contigs from the input assemblies are clustered based on pairwise Mash distances between contigs and the user must decide which clusters are valid. **(A)** presents FastME tree, built using the pairwise distances of contigs for BF5_0283 sample. Selected clusters (1, 2 and 9) meet the criteria: cluster contain many contigs (ideally 12, one from each assembly), contigs are close to each other and have similar lengths, and similar and realistic read depths. **(B)** presents FastME tree, built using the pairwise distances for Mt009 sample. Clusters were not well defined; selected clusters (1, 2, 3) only partly meet criteria used for BF5_0283.

A

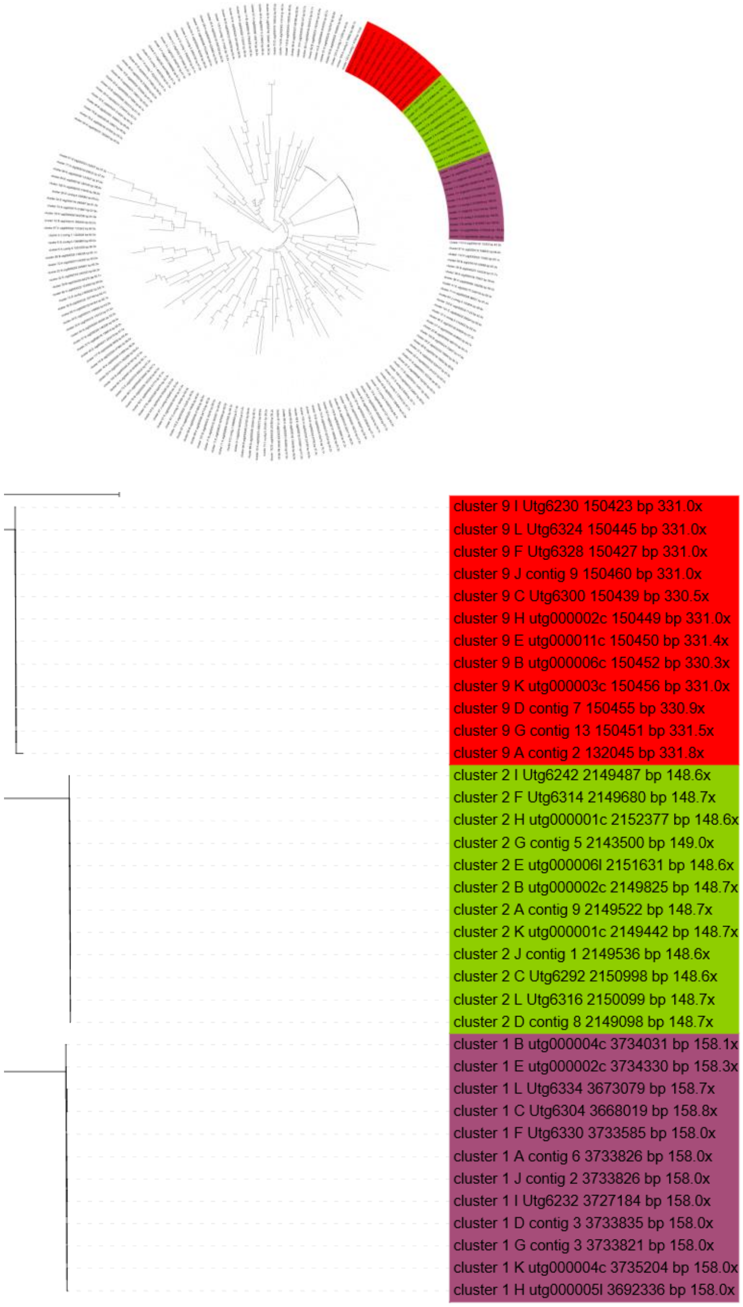

B

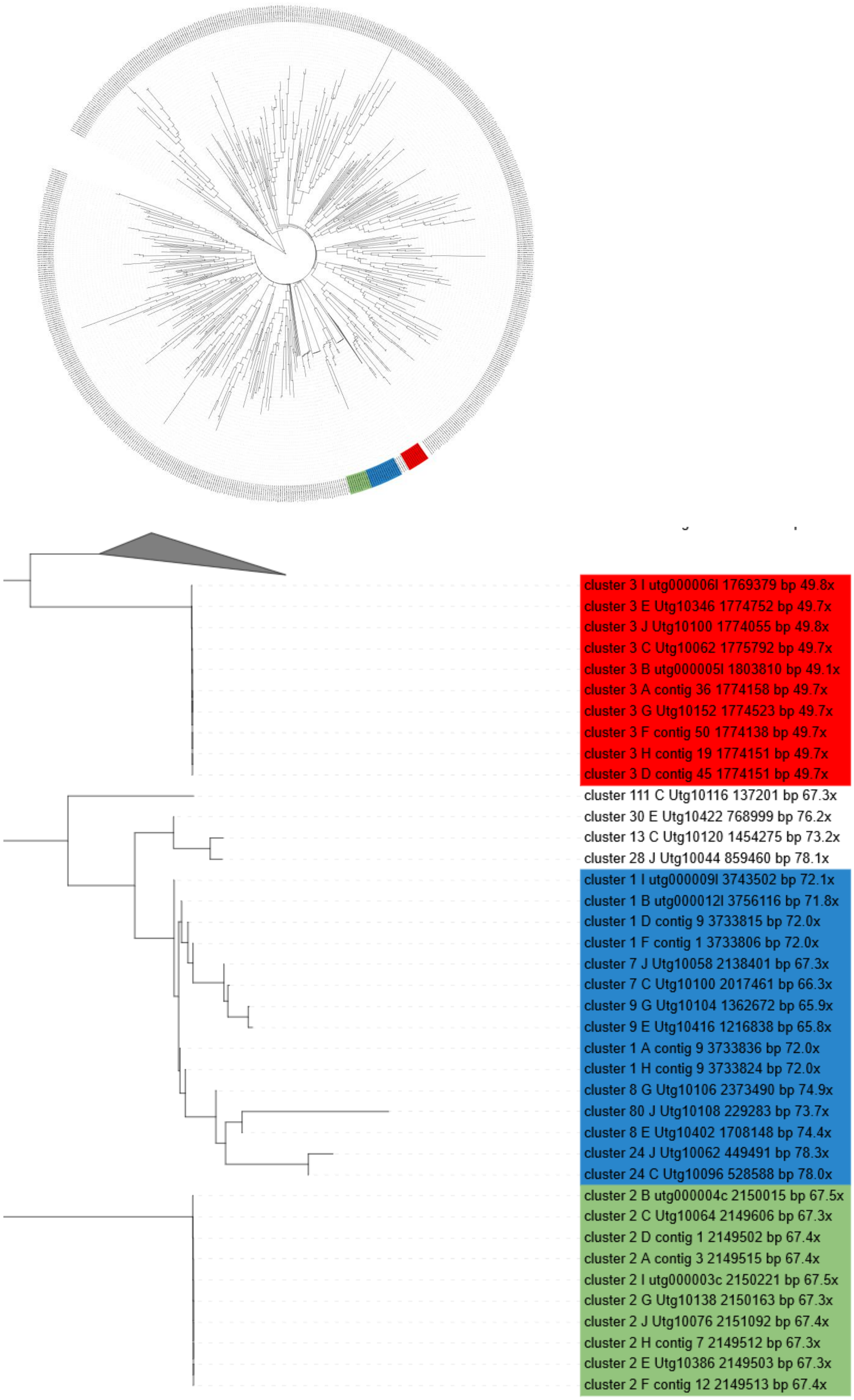

cluster 3 I utg000006l 1769379 bp 49.8x
cluster 3 E Utg10346 1774752 bp 49.7x
cluster 3 J Utg10100 1774055 bp 49.8x
cluster 3 C Utg10062 1775792 bp 49.7x
cluster 3 B utg000005l 1803810 bp 49.1x
cluster 3 A contig 36 1774158 bp 49.7x
cluster 3 G Utg10152 1774523 bp 49.7x
cluster 3 F contig 50 1774138 bp 49.7x
cluster 3 H contig 19 1774151 bp 49.7x
cluster 3 D contig 45 1774151 bp 49.7x
cluster 111 C Utg10116 137201 bp 67.3x
cluster 30 E Utg10422 768999 bp 76.2x
cluster 13 C Utg10120 1454275 bp 73.2x
cluster 28 J Utg10044 859460 bp 78.1x
cluster 1 I utg000009l 3743502 bp 72.1x
cluster 1 B utg000012l 3756116 bp 71.8x
cluster 1 D contig 9 3733815 bp 72.0x
cluster 1 F contig 1 3733806 bp 72.0x
cluster 7 J Utg10058 2138401 bp 67.3x
cluster 7 C Utg10100 2017461 bp 66.3x
cluster 9 G Utg10104 1362672 bp 65.9x
cluster 9 E Utg10416 1216838 bp 65.8x
cluster 1 A contig 9 3733836 bp 72.0x
cluster 1 H contig 9 3733824 bp 72.0x
cluster 8 G Utg10106 2373490 bp 74.9x
cluster 80 J Utg10108 229283 bp 73.7x
cluster 8 E Utg10402 1708148 bp 74.4x
cluster 24 J Utg10062 449491 bp 78.3x
cluster 24 C Utg10096 528588 bp 78.0x
cluster 2 B utg000004c 2150015 bp 67.5x
cluster 2 C Utg10064 2149606 bp 67.3x
cluster 2 D contig 1 2149502 bp 67.4x
cluster 2 A contig 3 2149515 bp 67.4x
cluster 2 I utg000003c 2150221 bp 67.5x
cluster 2 G Utg10138 2150163 bp 67.3x
cluster 2 J Utg10076 2151092 bp 67.4x
cluster 2 H contig 7 2149512 bp 67.3x
cluster 2 E Utg10386 2149503 bp 67.3x
cluster 2 F contig 12 2149513 bp 67.4x

Figure S2: Graphical presentation of contigs from Mt009 assemblies along with associated data with "anvi-interactive" function. **(A)** presents Trycycler assembly, **(B)** presents Unicycler assembly and **(C)** presents metaFlye assembly. Tree in the middle shows contigs presented in assemblies. Bins in **(B)** were manually refined based on differences in mean coverage (mapping of Illumina short reads), differences in GC content and genes taxonomy.

A.

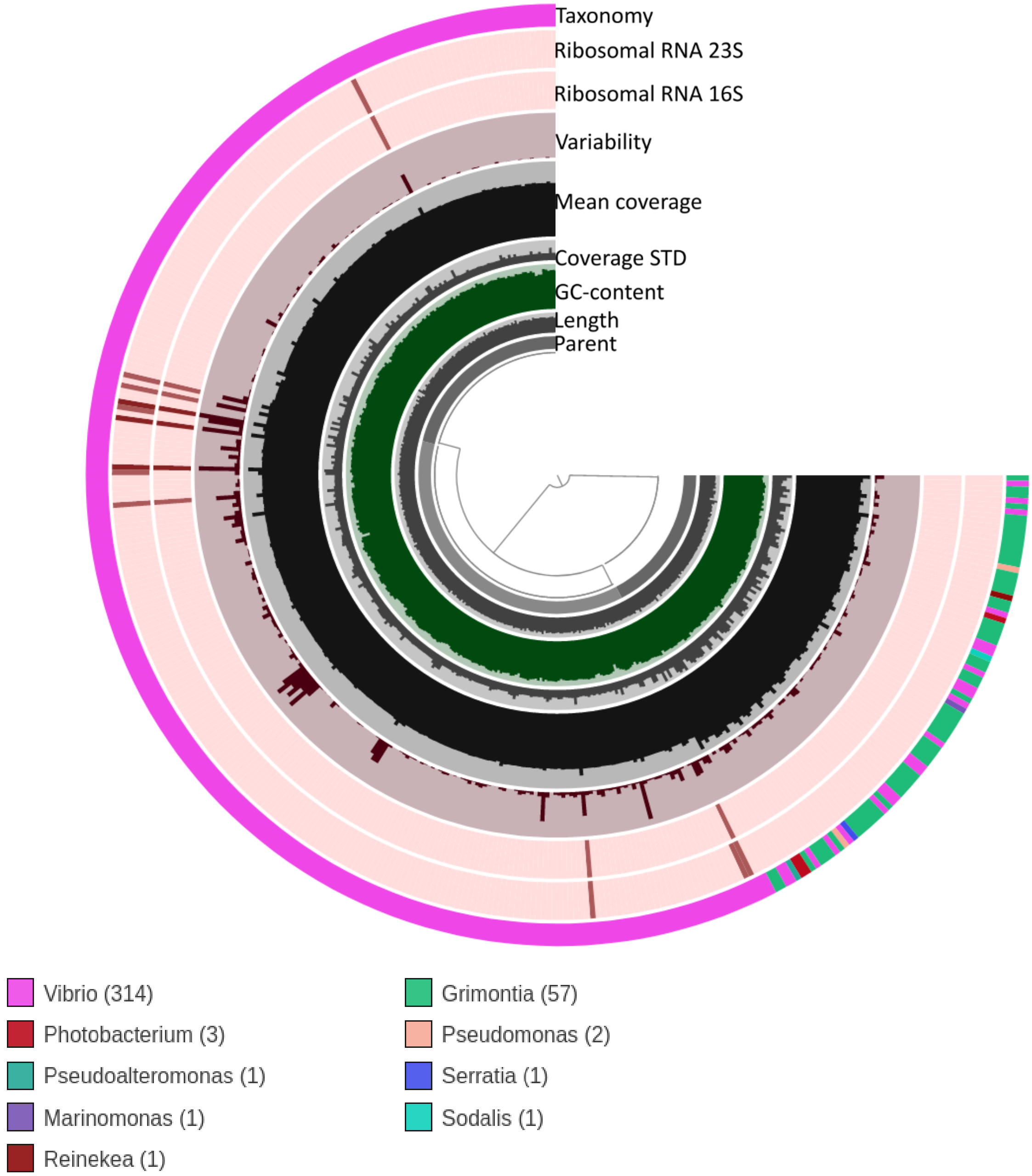

B.

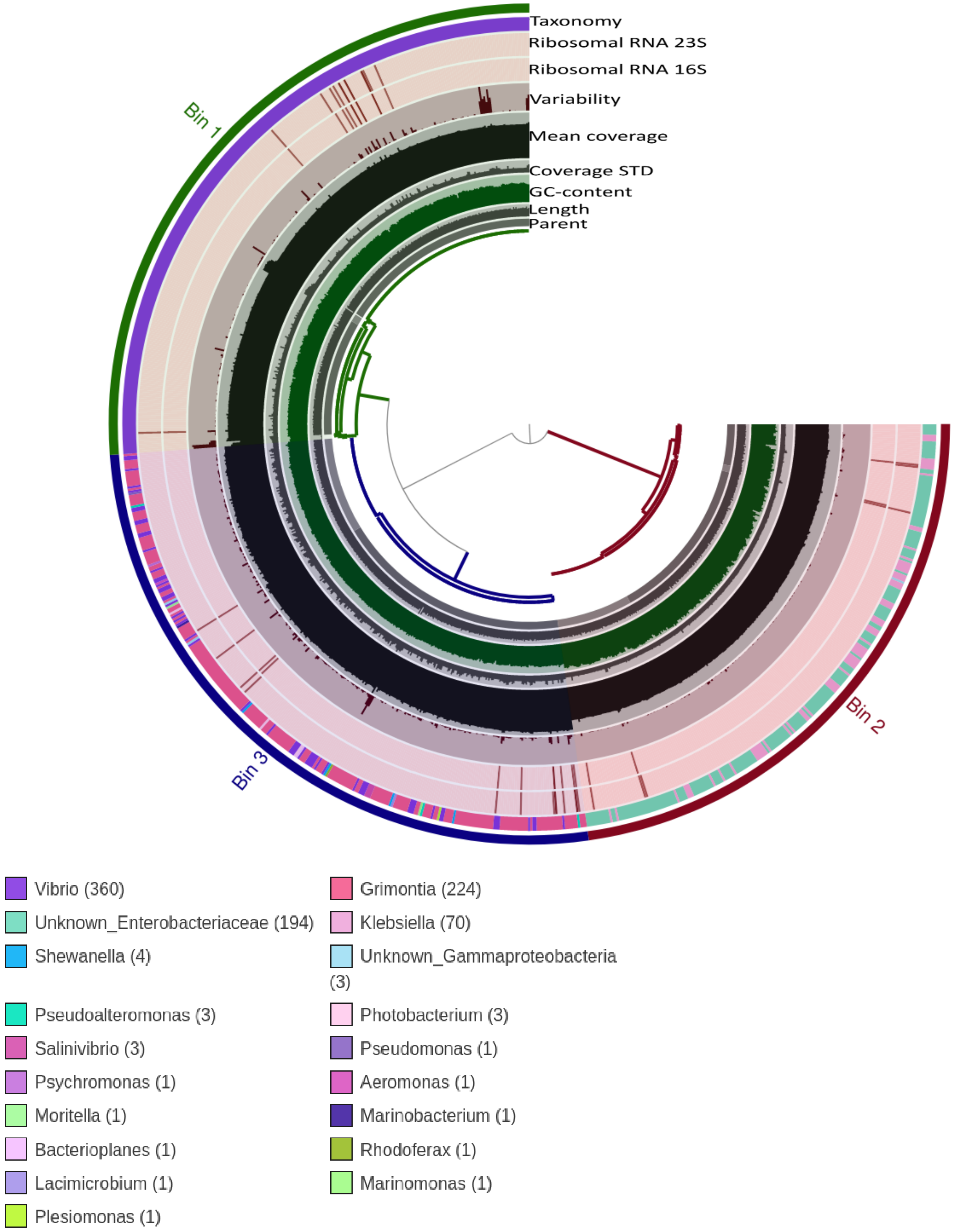

Taxonomy
Ribosomal RNA 23S
Ribosomal RNA 16S
Variability
Mean coverage
Coverage STD
GC-content
Length
Parent
Bin 1
Bin 2
Bin 3
Vibrio (360)
Grimontia (224)
Unknown_Enterobacteriaceae (194)
Klebsiella (70)
Shewanella (4)
Unknown_Gammaproteobacteria (3)
Pseudoalteromonas (3)
Photobacterium (3)
Salinivibrio (3)
Pseudomonas (1)
Psychromonas (1)
Aeromonas (1)
Moritella (1)
Marinobacterium (1)
Bacterioplanes (1)
Rhodoferax (1)
Lacimicrobium (1)
Marinomonas (1)
Plesiomonas (1)

C.

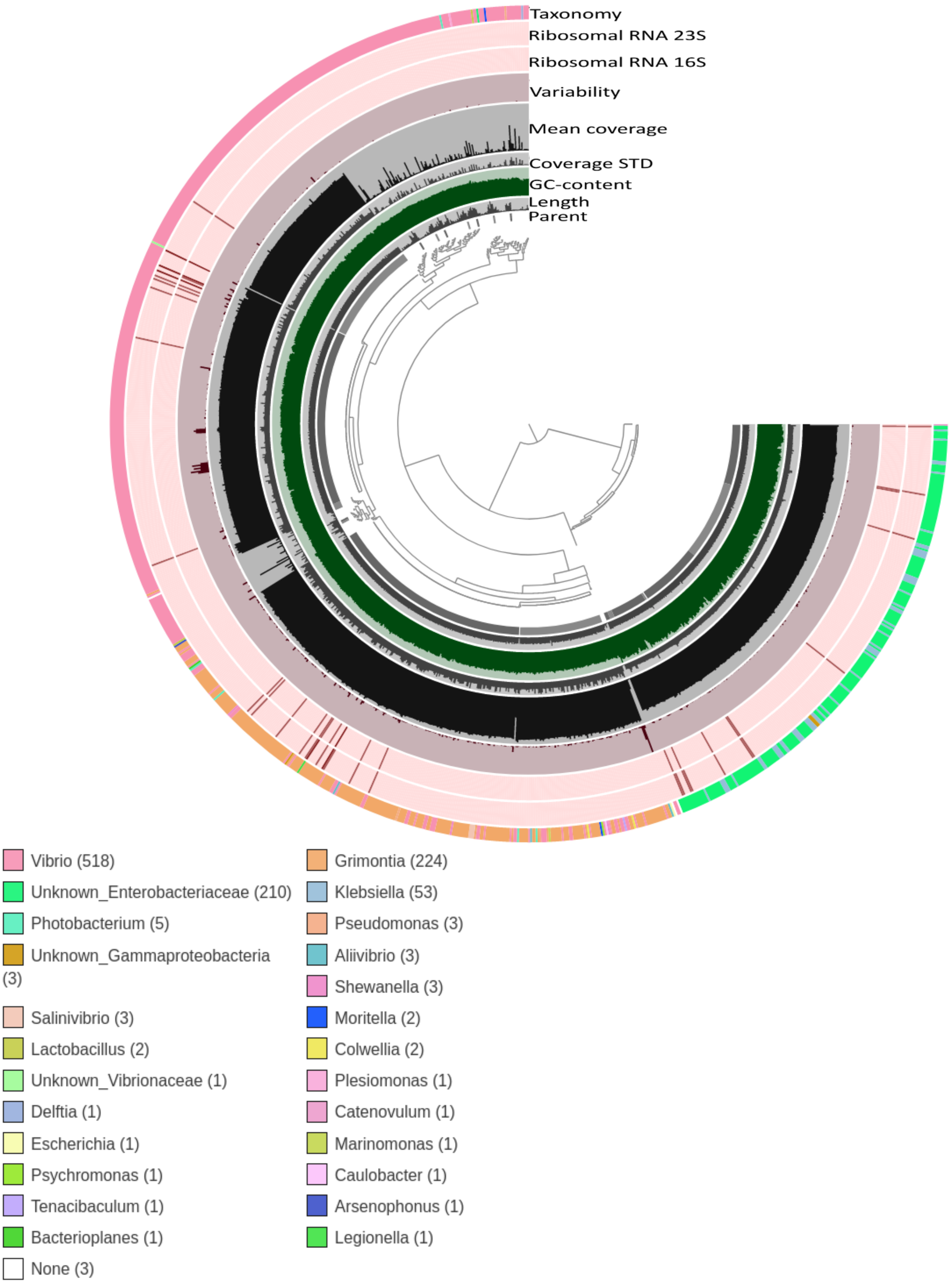
Taxonomy
Ribosomal RNA 23S
Ribosomal RNA 16S
Variability
Mean coverage
Coverage STD
GC-content
Length
Parent
Vibrio (518)
Grimontia (224)
Unknown_Enterobacteriaceae (210)
Klebsiella (53)
Photobacterium (5)
Pseudomonas (3)
Unknown_Gammaproteobacteria (3)
Aliivibrio (3)
Shewanella (3)
Salinivibrio (3)
Moritella (2)
Lactobacillus (2)
Colwellia (2)
Unknown_Vibrionaceae (1)
Plesiomonas (1)
Delftia (1)
Catenovulum (1)
Escherichia (1)
Marinomonas (1)
Psychromonas (1)
Caulobacter (1)
Tenacibaculum (1)
Arsenophonus (1)
Bacterioplanes (1)
Legionella (1)
None (3)

Figure S3: Results of PCR reaction with taxa-specific primers. Agarose gel electrophoresis of amplicons visualized by ethidium bromide staining and UV light. Samples on the gel are 1kb ladder, C- (negative control of PCR reaction), BF5_0283, BF5_0283, Mt009, Mt009, Mt009, C+ (positive control of PCR reaction), and C2- (negative control of DNA extraction). All samples were tested with (**A)** 16S universal primers, *V. campbellii* and (**B)** *K. pneumoniae* taxa-specific primers.

A.

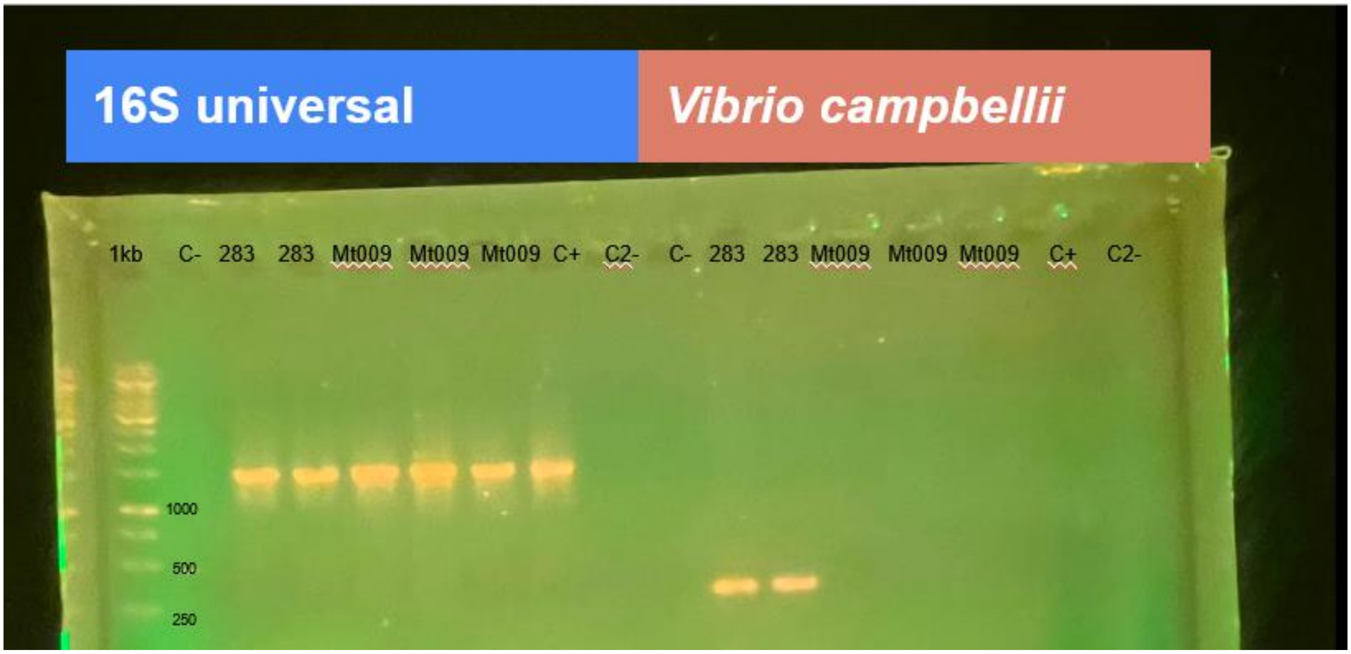

B.

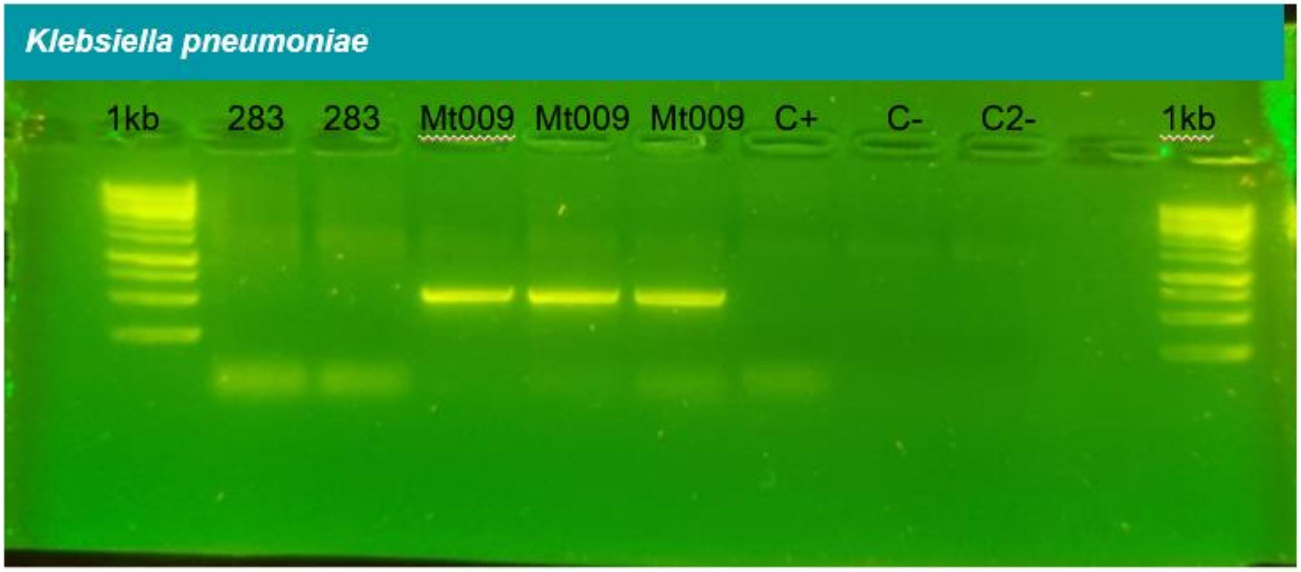

Figure S4: Number of genes assigned to the COG category on chromosomes (ChrI, ChrII) and the plasmid (P), presented with two bars corresponding to the two assemblies of the *V. campbellii* BF5_0283 genome. D, Cell cycle control and mitosis; M, Cell wall/membrane/envelope biogenesis; N, Cell motility; O, Post-translational modification, protein turnover, and chaperones; T, Signal transduction; U, Intracellular trafficking, secretion, and vesicular transport; V, Defense mechanisms; W, Extracellular structures; Z, Cytoskeleton; A, RNA processing and modification; J, Translation, ribosomal structure and biogenesis**;** K, Transcription**;** L, Replication and repair; C, Energy production and conversion; E, Amino acid metabolism and transport; F, Nucleotide metabolism and transport; G, Carbohydrate metabolism and transport**;** H, Coenzyme transport and metabolism; I, Lipid transport and metabolism; P, Inorganic ion transport and metabolism; Q, Secondary metabolites biosynthesis, transport and catabolism; R, General functional prediction only; S, Function unknown; X, Mobilome: prophages, transposons. .

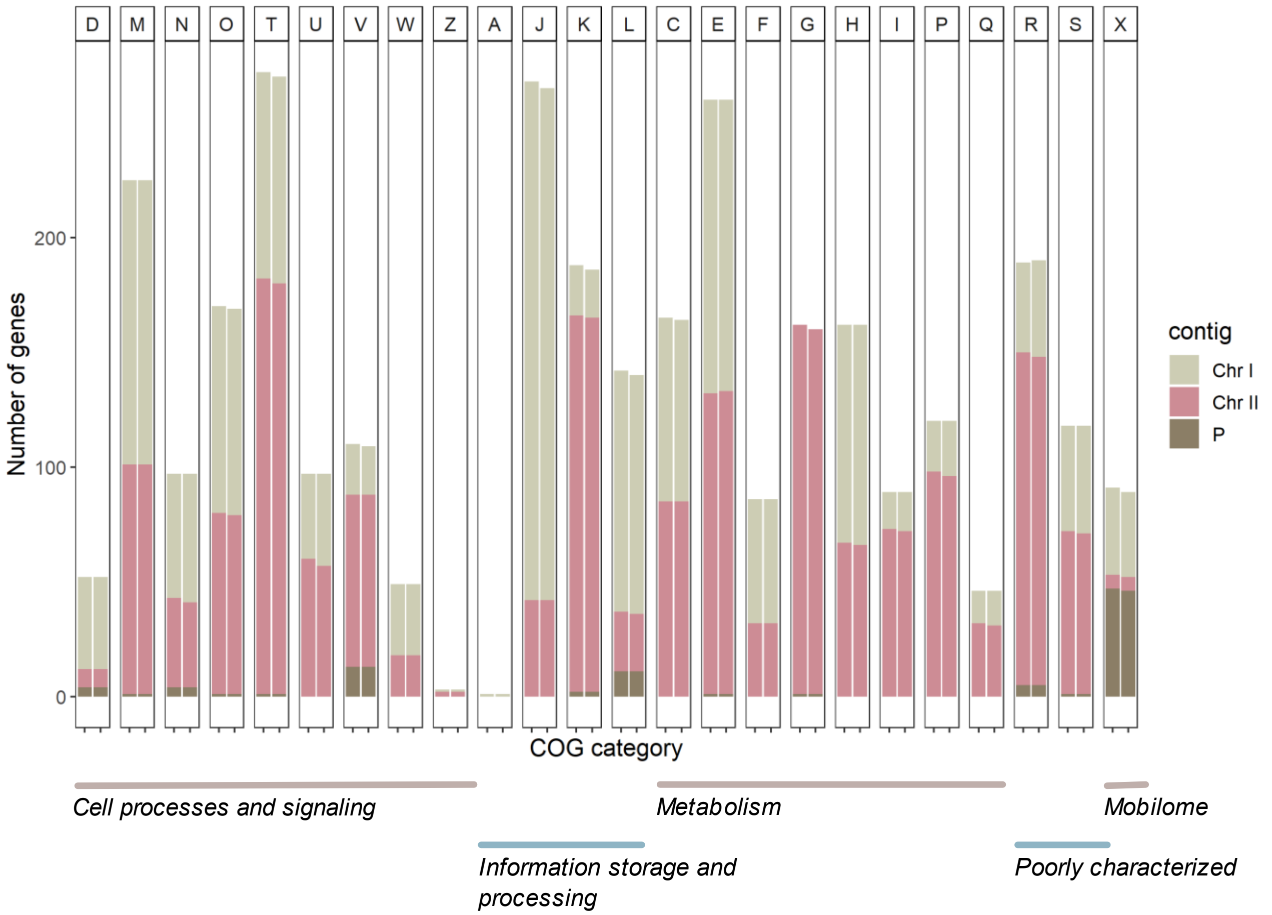

Figure S5: Gene abundance heat map (white = absent, green = high), representing abundance of genes in *V. campbelli* genomes, belonging to different COG categories. **(A)** presents annotations of genes in core collection (present in all genomes according to pangenome analyses), **(B)** presents annotations of genes in accessory Group 1 and Group 2 collections and **(C)** presents annotations of genes unique for BF_0283 and Mt009_b1.

A

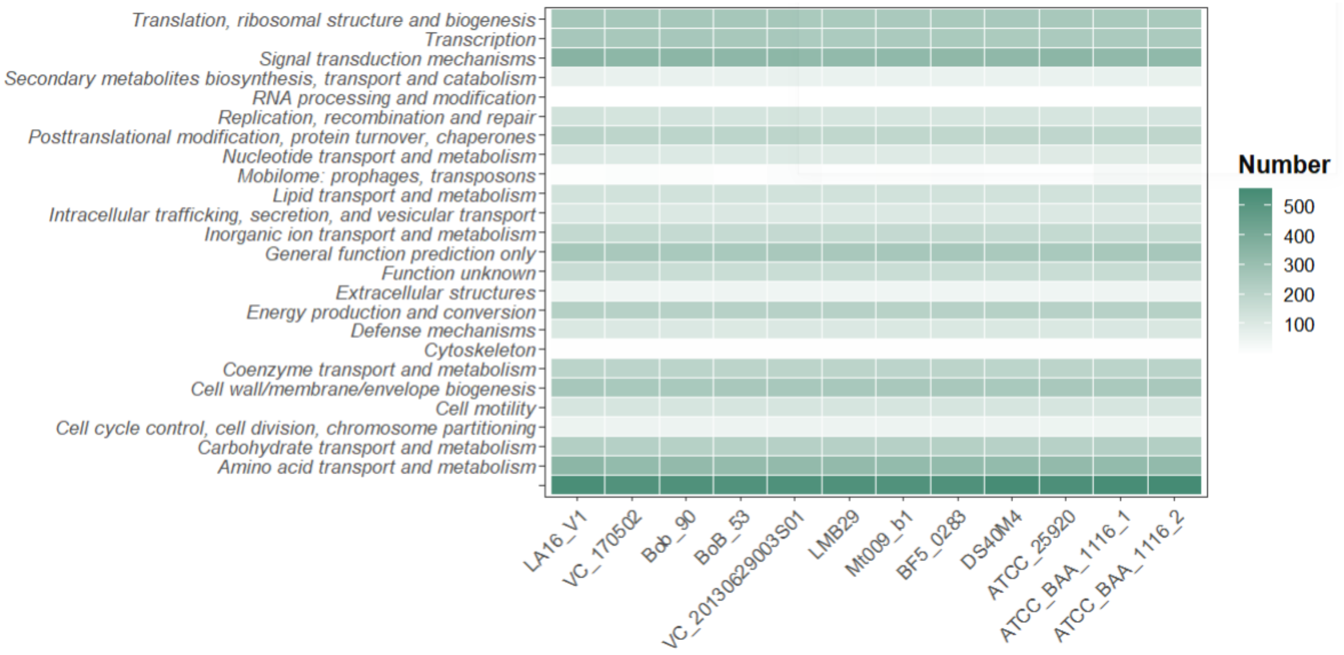

B

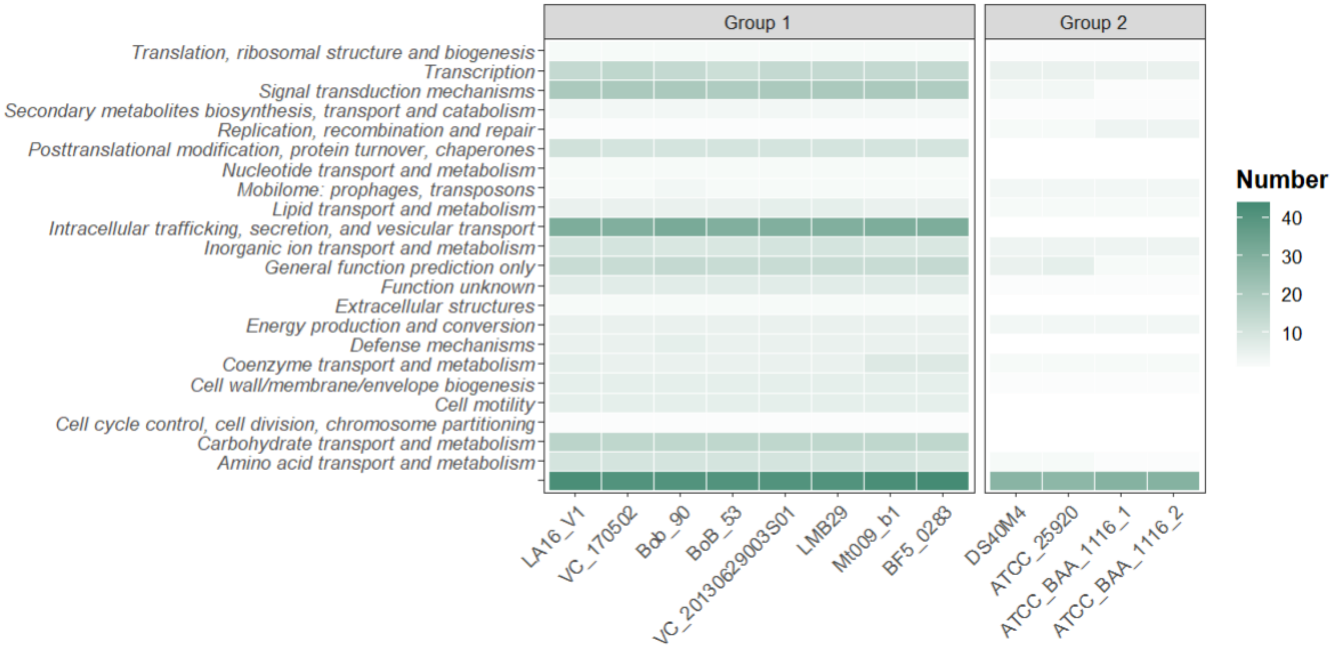
Group 1
Group 2
Translation, ribosomal structure and biogenesis
Transcription
Signal transduction mechanisms
Secondary metabolites biosynthesis, transport and catabolism
Replication, recombination and repair
Posttranslational modification, protein turnover, chaperones
Nucleotide transport and metabolism
Mobilome: prophages, transposons
Lipid transport and metabolism
Intracellular trafficking, secretion, and vesicular transport
Inorganic ion transport and metabolism
General function prediction only
Function unknown
Extracellular structures
Energy production and conversion
Defense mechanisms
Coenzyme transport and metabolism
Cell wall/membrane/envelope biogenesis
Cell motility
Cell cycle control, cell division, chromosome partitioning
Carbohydrate transport and metabolism
Amino acid transport and metabolism
LA16_V1
VC_170502
Bob_90
BoB_53
VC_20130629003S01
LMB29
Mt009_b1
BF5_0283
DS40M4
ATCC_25920
ATCC_BAA_1116_1
ATCC_BAA_1116_2
Number
40
30
20
10

C

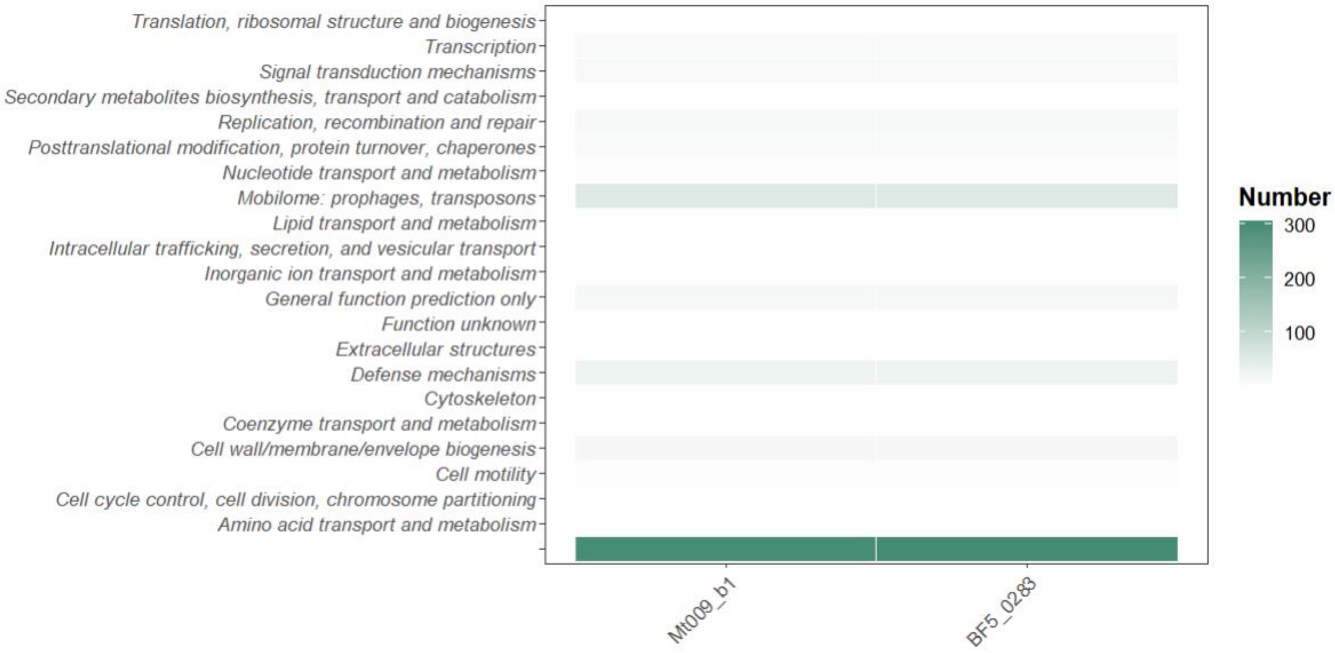
Translation, ribosomal structure and biogenesis
Transcription
Signal transduction mechanisms
Secondary metabolites biosynthesis, transport and catabolism
Replication, recombination and repair
Posttranslational modification, protein turnover, chaperones
Nucleotide transport and metabolism
Mobilome: prophages, transposons
Lipid transport and metabolism
Intracellular trafficking, secretion, and vesicular transport
Inorganic ion transport and metabolism
General function prediction only
Function unknown
Extracellular structures
Defense mechanisms
Cytoskeleton
Coenzyme transport and metabolism
Cell wall/membrane/envelope biogenesis
Cell motility
Cell cycle control, cell division, chromosome partitioning
Amino acid transport and metabolism
Mt009_b1
BF5_0283
Number
300
200
100
